# Establishment of a Prognostic Necroptosis-Related lncRNA Signature in Ovarian Cancer

**DOI:** 10.2174/0113862073339602241028095015

**Published:** 2025-01-07

**Authors:** Hui Xu, Meng Li, Wen-lan Qiao, Tian Hua

**Affiliations:** 1 Department of Gynecology, Affiliated Xingtai People Hospital of Hebei Medical University, Hebei, China

**Keywords:** Ovarian cancer, necroptosis-related long non-coding RNAs, prognostic model, immune response, qRT-PCR

## Abstract

**Introduction:**

Ovarian Cancer (OC) was known for its high mortality rate among gynecological malignancies, often resulting in a poor prognosis. This study sought to identify prognostic necroptosis-related long non-coding RNAs (lncRNAs) (NRlncRNAs) with prognostic potential and to construct a reliable risk prediction model for OC patients.

**Methods:**

The transcriptome and clinic data were sourced from TCGA and GTEx databases. Initially, NRlncRNAs were discovered by assessing gene correlations and evaluating differences in gene expression. Subsequently, Cox regression and LASSO methods were employed to develop the NRlncRNAs risk model, which was further validated through survival analysis, ROC curves, Cox regression, and nomograms across both the test and entire datasets.

**Results:**

Multivariate Cox analysis revealed that the risk score based on 14 NRlncRNAs can independently predict the prognosis of OC. The low-risk group demonstrated significantly higher immune cell infiltration scores and lower tumor immune dysfunction, exclusion, and TIDE scores, as well as an increased number of neoantigens and higher TMB. Notably, the low-risk group also exhibited an elevated HRD score.

**Conclusion:**

The model’s predictive accuracy was further substantiated through ROC analysis, showing superior performance compared to many existing models.Finally, the expression levels of 14 NRlncRNAs were confirmed using the qRT-PCR in two OC cell lines. These findings suggested that the NRlncRNAs risk model could serve as a more precise indicator for forecasting immune response and outcomes of targeted treatments in OC.

## 1 INTRODUCTION

Ovarian Cancer (OC) was known for having the bleakest prognosis among gynecologic malignancies and ranked as the fifth leading cause of cancer-related mortality in women. [1]. Over the past several decades, fewer than 45% of OC patients have survived longer than five years [2]. A significant contributor to the high death rate from this disease was that about 70% of patients were initially diagnosed at an advanced stage [3]. The first-line treatment strategy for OC was 3-6 courses of platinum-taxanes combination chemotherapy following the primary debulking surgery [4]. However, most advanced-stage OC patients would suffer from the recurrence of the disease within two years, often accompanied by the development of platinum resistance, leading to death [5]. Therefore, reliable biomarkers in OC have garnered increasing attention to facilitate early diagnosis, the monitoring of treatment response, and the detection of disease recurrence.

Necroptosis was a form of regulated non-cysteine protease-dependent programmed cell death that exhibits morphological features similar to necrosis. This process operated through a unique mechanism distinct from the apoptotic signaling pathway. Also known as programmed necrosis, necroptosis mechanistically resembles apoptosis while morphologically resembling necrosis. The initiation of this regulated necrosis primarily depended on the kinase activity of receptor-interacting protein kinase 3 (RIPK3). RIPK3 interacted with other RHIM-containing proteins, such as RIPK1, TRIF, or ZBP1, depending on the specific stimuli present during infection and inflammation. This regulated form of cell death played a significant role in various physiological and pathological processes [6-8]. Necroptosis caused wide concern due to the emerging effects on the modulation of cancer initiation, development, and metastasis issues [9]. Liu *et al.* [10] revealed that genes linked to necroptosis significantly influenced the tumorigenic potential of cancer cells and their resistance to radiotherapy, offering strong evidence for the crucial role of necroptotic signaling in cancer. Prior research has suggested that the expression of necroptosis-related genes may correlate with clinical outcomes in different malignancies [11-13]. Recent studies have created a model utilizing necroptosis-related genes to forecast the prognosis of patients with advanced OC [14].

Consequently, a comprehensive investigation of the regulatory network underlying necroptosis was imperative to devise a new strategy for modulating necroptosis in OC. LncRNAs represented a category of transcripts that were over 200 nucleotides long. Recent research has revealed that lncRNAs play a role in numerous essential biological activities, including tumor formation, cancer progression, and immune response [15, 16]. Several studies have explored the function of necroptosis-related lncRNAs (NRlncRNAs) across various cancer types [17, 18]. Recent research indicated that NRlncRNAs may be used to forecast prognosis and assist in identifying cold *versus* hot tumors in gastric cancer [19]. Wang *et al.* discovered a necroptosis-associated prognosis marker for gastric cancer [20]. However, the role of NRlncRNAs has not been clearly described in OC. Therefore, exploring the roles of NRlncRNAs was essential in predicting prognosis and individual immune-associated therapy formulated in OC.

In our research, we sought to establish a robust NRlncRNAs model associated with prognosis in OC. The model’s sensitivity and specificity were assessed using ROC analysis and benchmarked against several previously published risk models. Using the risk score, we conducted further studies, including Kaplan-Meier analysis, gene set enrichment analyses (GSEA), and the assessments of correlation with the tumor immune evasion, TIDE level, neoantigen level, TMB, immune filtration score, and HRD status.

## 2 METHODS AND MATERIALS

### 2.1 Dataset Collection

The transcriptional profiles and associated clinical data for 379 ovarian cancer (OC) patients were obtained from the Xena browser linked to TCGA [21]. Additionally, due to the absence of normal OC sample transcriptional profiles, we downloaded the transcriptional data of 88 normal human ovarian samples from the Xena browser associated with the GTEx project.

### 2.2 Identification of Differentially Expressed NRlncRNAs and Development of the Prognostic Model

Based on prior studies on necroptosis, we identified 67 necroptosis-related genes (NRGs) [19]. We filtered all lncRNAs using the GRCh38.p13 GTF file. Subsequently, we employed R to compute the Pearson correlation between lncRNAs and NRGs, identifying 385 NRlncRNAs that satisfied the stringent thresholds of *p* < 0.001 and correlation strength > 0.4 Through the Wilcoxon test, 215 NRlncRNAs were identified as differentially expressed between the TCGA and GTEx cohorts, meeting the criteria of |Log2 fold change| > 1 and FDR < 0.05. Following this, the data was split into training and testing sets in equal proportions (1:1) by R package “caret”. All the subsequent analyses were conducted in both train, test, and entire dataset. After performing the use of univariate Cox regression to evaluate gene correlations and survival outcomes in the TCGA cohort, 30 NRlncRNAs were identified (R package “survival”). LASSO regression with 10-fold cross-validation was applied to prevent overfitting (R package “glmnet”). Subsequently, multivariate Cox regression was executed to compute the coefficient and further streamline the model’s variables, ultimately identifying 14 NRlncRNAs for the final risk model. The risk score for each patient was determined using the specified formula: (NRlncRNA 1 expression×coef) + (NRlncRNA 2 expression×coef) + . . . + (NRlncRNA n expression×coef). OC patients were separated into high-risk and low-risk groups according to their median scores. The R package “timeROC” was employed to perform the time-dependent ROC curve analysis.

### 2.3 Evaluation of the Prognostic Model by Nomogram, Calibration

Using the R package “rms,” we built a nomogram using the multivariate Cox regression analysis of clinical data and risk scores. Calibration curves at 1-year, 3-year, and 5-year intervals were then employed to assess the nomogram’s accuracy.

### 2.4 Functional Pathways Analysis

GSEA v4.3.2 was obtained from the MSigDB database [22, 23] to examine the tightly related GO and KEGG pathways. The criteria used for selection were *p* < 0.05, FDR < 0.25, |NES| > 1.5.

### 2.5 Analysis of ssGSEA, Immune Infiltration

Thirteen immune-related pathways and 16 immune cells were obtained to explore the immune features difference between low-risk and high-risk subgroups through GSVA and GSEABase. To ensure a robust evaluation of the immune infiltration status, we downloaded a curated file (including cell type quantification methods of CIBERSORT, TIMER, QUANTISEQ, EPIC, XCELL, CIBERSORT-ABS, and MCPCOUNTER) from TIMER2.0 [24]. Spearman correlation analysis was then applied to investigate the relationship between immune infiltration scores and risk scores.

### 2.6 Calculation of TMB, TIDE, NEOANTIGEN, and HRD SCORES

The TIDE platform was utilized to calculate tumor immune dysfunction, exclusion, and TIDE score [25]. TMB and HRD were calculated in our previous study [26]. Neoantigen data was obtained from the Cancer Immunome Atlas [27]. To assess the differences between low-risk and high-risk subgroups, we employed the Wilcoxon test. T cell exhaustion and T cell regulatory genesets were obtained from IOBR [28].

### 2.7 qRT-PCR

SKOV3, A2780, ISOE cell lines were purchased from the American Type Culture Collection (ATCC, Manassas, VA, the USA), the media for all the cell lines were supplemented with 10% fetal bovine serum (FBS, Gibco, CA, the USA), and 1% streptomycin/penicillin (New Cell & Molecular Biotech, China), with cells cultured in a 5% CO_2_ incubator at 37°C. RNA extraction from cell lines was performed with TRIzol reagent (Generay Biotech, Shanghai, Co., Ltd., China). To synthesize cDNA, 500 ng of total RNA was used with the Revert-Aid First Strand cDNA Synthesis Kit (Thermo Scientific, USA). For reverse transcription quantitative PCR, the QuantiNova TMSYBR^®^ Green PCR Kit (Qiagen, Hilden, Germany) was utilized. The primer sequences of 14 NRlncRNAs were obtained from Sangon Biotech Co. Ltd. (Table **S1**).

### 2.8 Statistical Analysis

Statistical analyses were carried out with the R software 4.1.1 A *p*-value of less than 0.05 was considered statistically significant. Unless otherwise noted, *, **, and *** indicate *p*-values of less than 0.05, 0.01, and 0.001, respectively. To compare survival curves, the log-rank test was employed to determine the Hazard ratio (HR) and *p*-value.

## 3 RESULTS

### 3.1 Identification of NRlncRNAs in OC

**Fig. (2):**
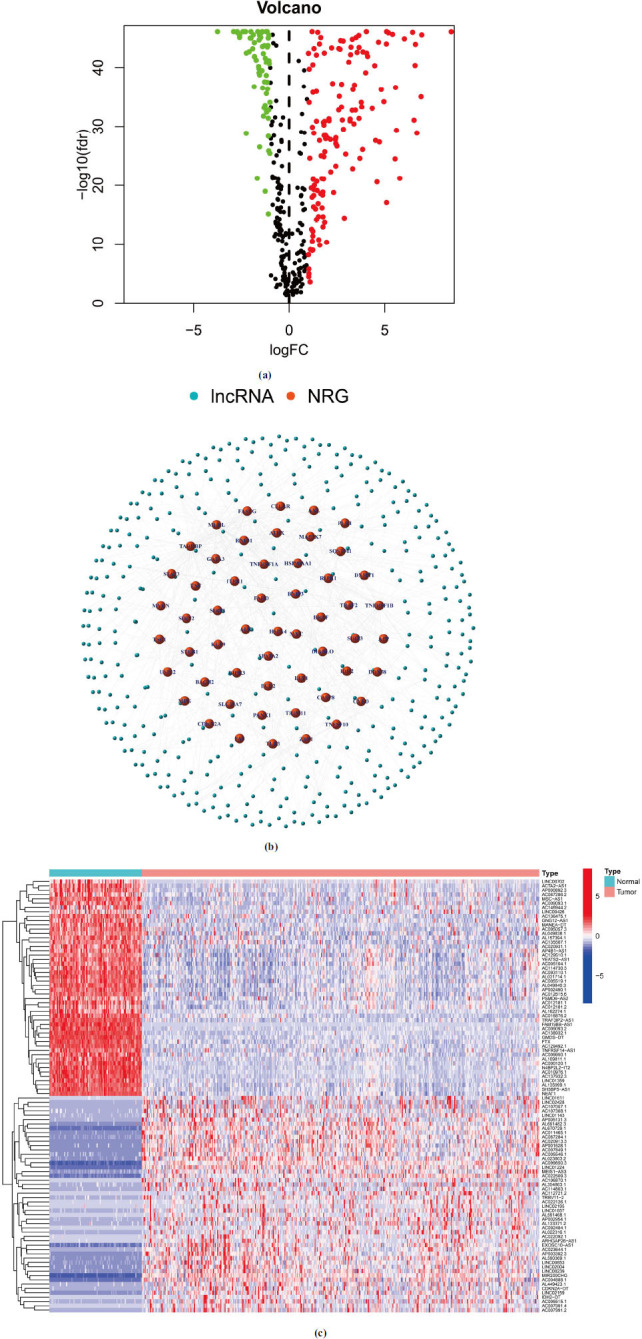
Identification of NRlncRNAs in OC patients. (**a**) Volcano plot showing genes with differential expression in necroptosis, with green points indicating significantly downregulated genes and red points indicating significantly upregulated genes. (**b**) Network illustrating the relationships between necroptosis genes and lncRNAs. (**c**) The top 50 NRlncRNAs with differential expression, ranked by log2 fold change comparing tumors and normal cases.

The study flow is illustrated in Fig. (**1**). A total of 67 NRGs were selected based on previous reports about necroptosis. Initially, 385 NRlncRNAs were through the correlation coefficients with 67 NRGs. After filtering for differential expression on the basis of |Log2 fold change| > 1 and FDR < 0.05, we obtained 215 NRlncRNAs. Among these, 146 NRlncRNAs were upregulated, while 69 were downregulated in OC tumors (Fig. **2a**). Fig. (**2b**) presented a network diagram and data that illustrated the relationships between NRGs and lncRNAs. The top 50 differentially expressed NRlncRNAs, ranked by Log2 fold change, are visualized in Fig. (**2c**).

### 3.2 Model Construction and Validation of NRlncRNAs in OC

**Fig. (3):**
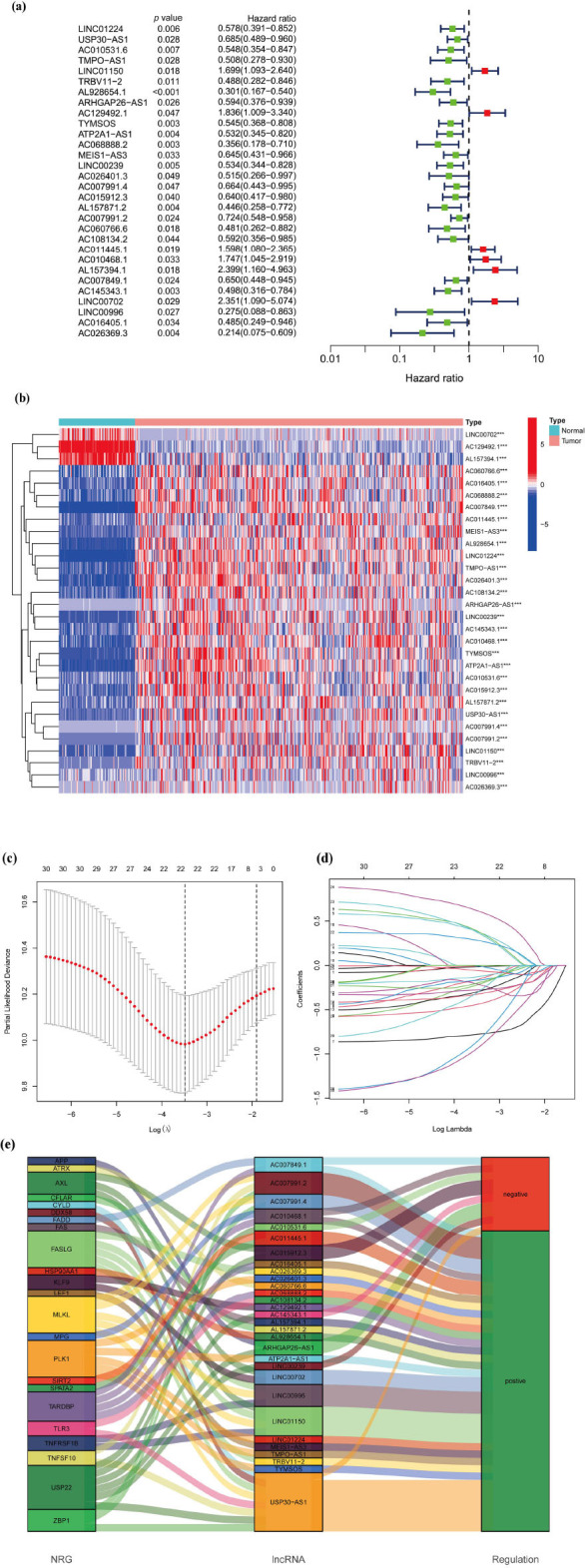
Development of the NRlncRNAs prognosis model in OC. (**a**) Prognostic lncRNAs identified using univariate Cox regression analysis. (**b**) Expression profiles of 30 prognostic NRlncRNAs. (**c**) Variable selection through 10-fold cross-validation in LASSO regression. (**d**) LASSO coefficient profile of NRlncRNAs. (**e**) Sankey plot illustrating the association between necroptosis genes and NRlncRNAs.

**Fig. (4):**
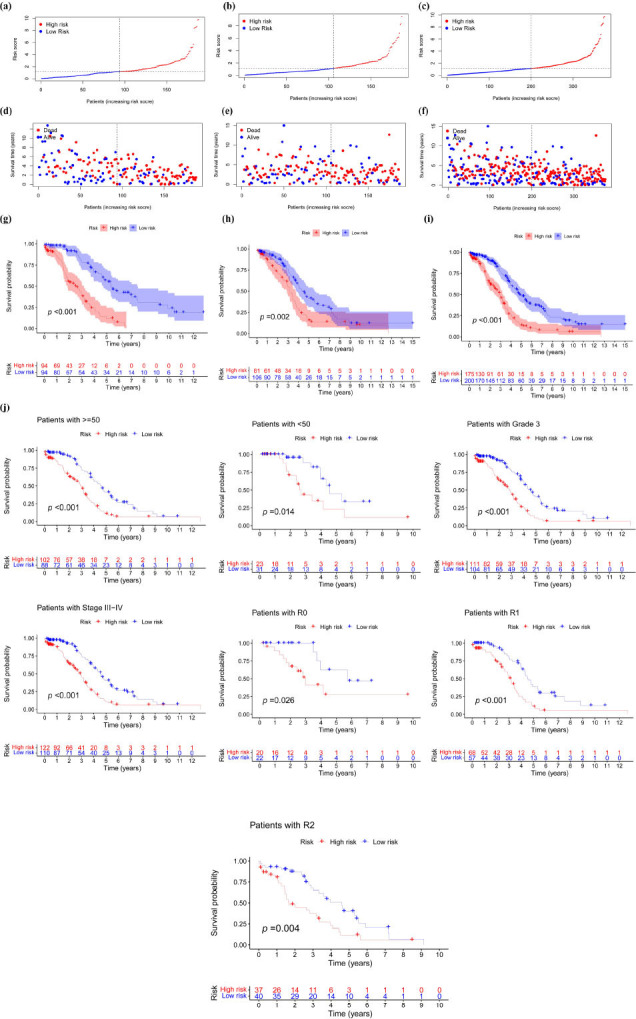
Prognosis of the 14 NRlncRNAs model across the train, test, and entire sets. (**a-c**) Risk score based presentation of the NRlncRNAs model across the train, test, and entire sets. (**d-f**) Survival comparisons between high-risk and low-risk subgroups across the train, test, and entire sets. (**g-i**) Survival curves for patients’ OS comparing between high-risk and low-risk subgroups across the train, test, and entire sets, respectively. (**j**) Survival curves demonstrating the prognostic significance of OS, divided by age, grade, stage, and tumor residual size, comparing high-risk and low-risk subgroups in the complete set.

Of the 215 NRlncRNAs, 30 were significantly correlated with OS in OC patients, as determined by univariate Cox regression (Fig. **3a**). A heatmap visualizing these 30 NRlncRNAs in Fig. (**3b**). LASSO regression was applied to the 30 NRlncRNAs, resulting in 23 NRlncRNAs when the first-rank value of Log(λ) exhibited the lowest deviance likelihood (Fig. **3c** and **d**). Additionally, a Sankey plot was created to visualize the interplay between the 30 NRlncRNAs and NRGs (Fig. **3e**). Furthermore, multivariate Cox regression was subsequently performed on the 23 NRlncRNAs, 14 NRlncRNAs were exacted finally. The risk score for each patient was determined using the specified formula:

Risk score = AC010531.6 × (-0.5433) + LINC01150 × (0.4659) + AL928654.1 × (-0.9543) + AC129492.1 × (0.4973) + MEIS1-AS3 × (-0.4623) + LINC00239 × (-0.4769) + AC007991.4 × (-0.4403) + AC060766.6 × (-0.6072) + AC011445.1 × (0.4920) + AC010468.1 × (0.5779) + AL157394.1 × (0.9963) + LINC00996 × (-1.5291) + AC016405.1 × (-0.7277) + AC026369.3 × (-1.6059). By applying the median risk score as a cutoff, OC patients were classified into high-risk and low-risk groups.s The risk score distribution was shown for the train, test, and entire datasets (Fig. **4a-c**). The demonstrations of high-risk and low-risk cases with survival status are shown in Fig. (**4d-f**). High-risk patients exhibited worse prognoses than low-risk patients, as illustrated in Figs. (**4g-i**). Additionally, even when factoring in age, stage, grade, and tumor residuals, the results remained consistent (Fig. **4j**).

### 3.3 Assessment and Validation of the Nomogram

**Fig. (5):**
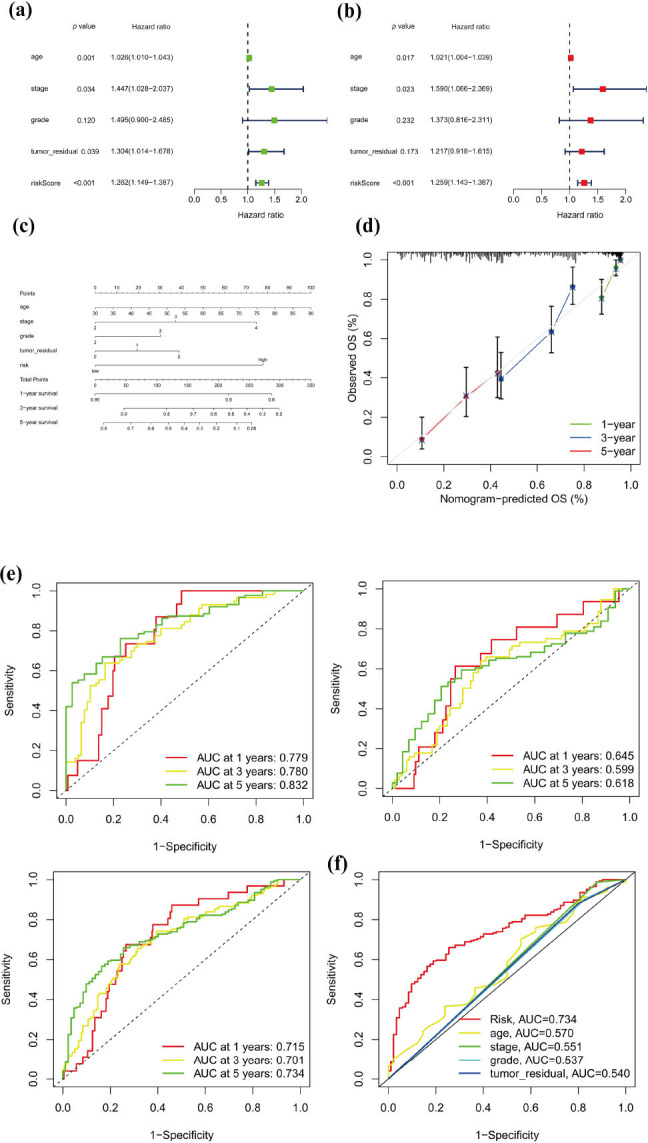
Nomogram and assessment of the risk model based on 14 NRlncRNAs. (**a-b**) Univariate and multivariate Cox regression analyses evaluating the relationship between the risk score, clinical features, and OS. (**c**) Nomogram that combined the risk score, age, grade, stage, and tumor residual size to estimate the probability of the OS at 1 year, 3 years, and 5 years. (**d**) Calibration plots for 1-, 3-, and 5-year OS predictions. (**e**) Time-dependent ROC curves for 1-, 3-, and 5-year predictions for the train, test, and entire sets. (**f**) ROC curves comparing the risk score with other clinical factors at 5-year.

To predict survival risk in OC patients, we constructed a nomogram using the complete TCGA dataset. The independent prognostic values of the risk score from the 14 NRlncRNAs were first assessed using univariate and multivariate Cox regression analyses, as well as patient age, stage of the disease, grade, and size of tumor residuals. The findings presented in Fig. (**5a-b**) demonstrated that the risk score from this model functioned as the independent predictor of prognosis for OC patients. A nomogram was subsequently developed to project the OS rates for OC patients at intervals of 1 year, 3 years, and 5 years (Fig. **5c**). Calibration plots for these time points were used to verify that the nomogram accurately predicted 1 year, 3 years, and 5 years OS (Fig. **5d**).

### 3.4 Risk Model Evaluation

The ROC was conducted to assess the risk model’s sensitivity and specificity on the prognosis. The results were presented using the AUC. The NRlncRNAs risk model achieved notable AUC of 0.779, 0.780, and 0.832 for 1 year, 3 years, and 5 years predictions in the training set, respectively. In the test set, the AUC was 0.645, 0.599, and 0.618 for the same intervals. For the entire dataset, the AUC was 0.715, 0.701, and 0.734 at 1 year, 3 years, and 5 years (Fig. **5e**), indicating that the NRlncRNAs risk signature effectively predicted survival. Additionally, at the five-year mark, the risk score's AUC of 0.734 outperformed other clinical features such as patient age (AUC = 0.570), stage (AUC = 0.551), grade (AUC = 0.537), and size of tumor residual (AUC = 0.540) (Fig. **5f**). We compared many risk models previously published in OC and found that our model had higher AUC values than theirs at 1 year, 3 years and 5 years, including a previously published 8 necroptosis-related lncRNAs model [29] (Fig. **S1a**), a 5 co-expression network lncRNAs model [30] (Fig. **S1b**), a 5 ferroptosis-related genes model [31] (Fig. **S1c**), a 5 regulator of G protein signaling (RGS) genes model [32] (Fig. **S1d**), a 5 immune-related genes model [33] (Fig. **S1e**), a 6 pyroptosis-related genes model [34] (Fig. **S1f**), a 6 invasion-related genes model [35] (Fig. **S1g**), a 7 tumor microenvironment (TME)-related genes model [36] (Fig. **S1h**), a 7 pyroptosis-related genes model [37] (Fig. **S1i**).

### 3.5 Investigation of Different Molecular Characteristics in Risk Subroups

**Fig. (6):**
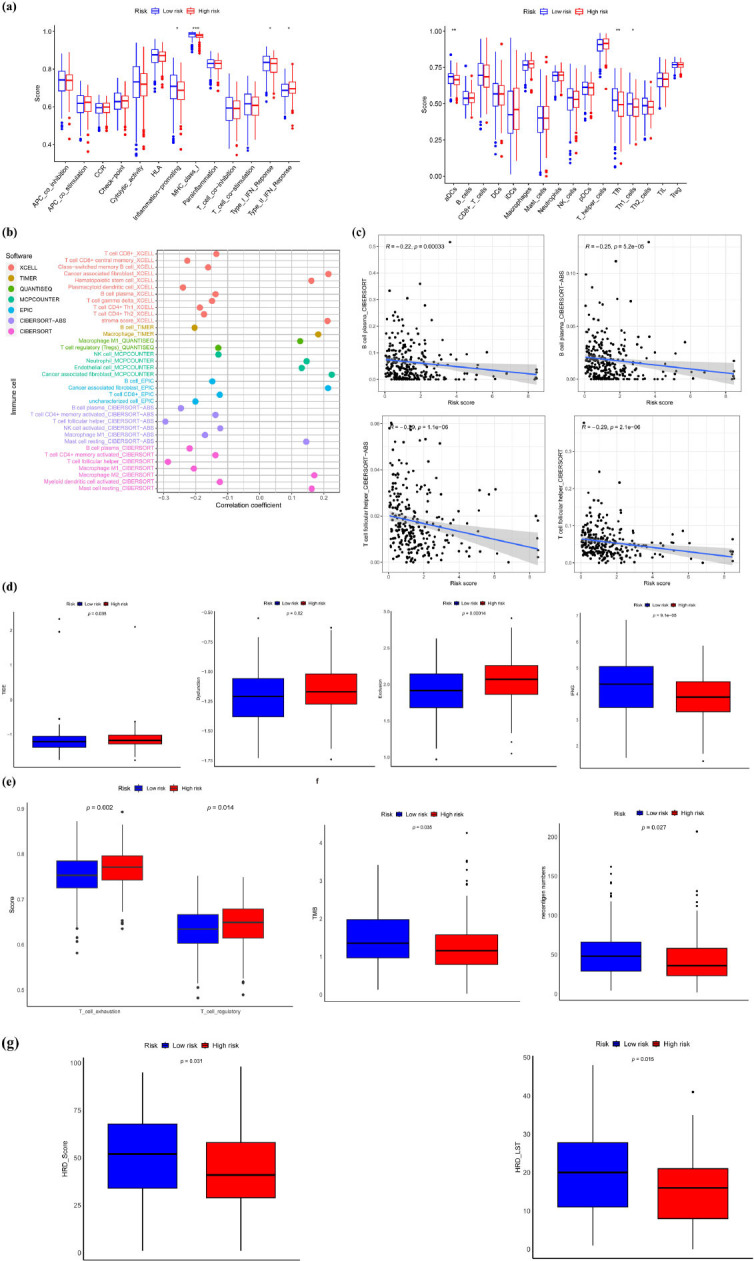
The investigation of different molecular characteristics across the high-risk and low-risk subgroups. (**a**) The levels of the immune function and cells in OC. (**b**) The immune cell bubble of risk grups. (**c**) Relationship across risk score and immune cell levels. (**d**) Boxplot representation of TIDE, dysfunction, exclusion, and IFNG scores in the high-risk group *versus* the low-risk group. (**e**) Boxplot representation of T cell exhaustion and T cell regulatory scores in the high-risk group *versus* the low-risk group. (**f**) The TMB and neoantigen numbers comparison across the high-risk and low-risk group. (**g**) The comparison of HRD and HRD_LST scores across the high-risk and low-risk group.

ssGSEA was used to compare enrichment scores for 13 immune-related pathways and 16 immune cell types between low-risk and high-risk groups. Some immune pathways and cells, such as MHC class I, Type I IFN Response, Tfh, and Th1 cells, had higher scores in the low-risk group than in the high-risk group (Fig. **6a**). Various algorithms further indicated an enhanced relationship of immune cells with the low-risk group, as shown in the immune cell bubble chart, such as T cell follicular helper, B cell plasma, Macrophage M1 by algorithm CIBERSORT-ABS, Plasmacytoid dendritic cell, T cell CD8+ central memory, T cell CD4+ Th1 by algorithm XCELL (Fig. **6b**, Table **S2**). Moreover, lower-risk scores were found to have a stronger association with immune cells, such as B cells and T follicular helper cells (Fig. **6c**). These results suggested that the low-risk group demonstrated increased immune infiltration. The high-risk group notably had increased TIDE, dysfunction, and exclusion scores, as well as reduced IFNG levels, in comparison with the low-risk group (Fig. **6d**). Additionally, we noted that the high-risk group exhibited higher T cell exhaustion and regulatory scores (Fig. **6e**), suggesting that patients in the low-risk group can be more responsive to immunotherapy treatments. Additionally, low-risk patients exhibited higher TMB levels compared to the high-risk group, aligning with the differential trend of neoantigen numbers between the two risk groups (Fig. **6f**). Significantly higher HRD and HRD_LST scores were observed in the low-risk group relative to the high-risk group (Fig. **6g**). The risk model can offer an alternative approach for selecting patients for therapy using poly ADP ribose polymerase (PARP) inhibitors.

**Fig. (7):**
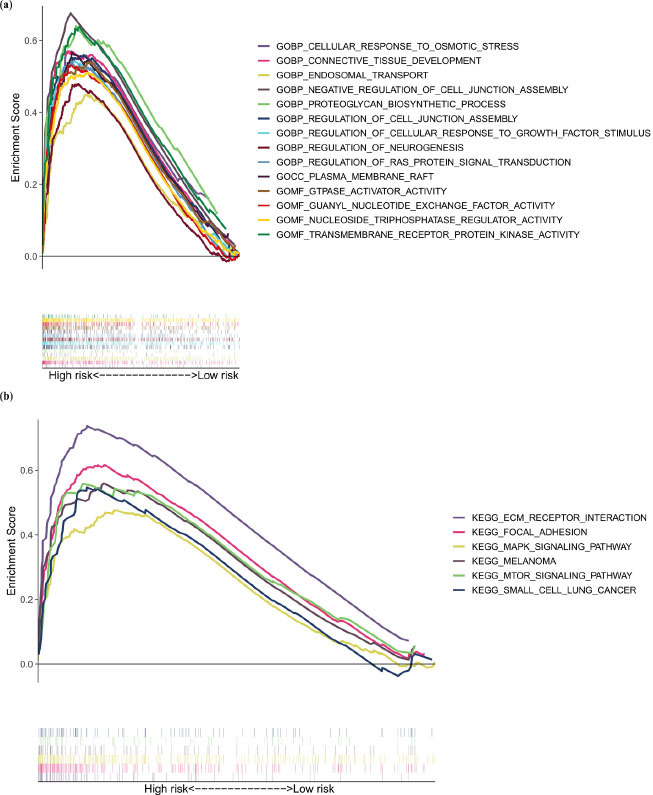
The investigation of different pathways enriched across the high-risk and low-risk subgroups. (**a**) GSEA of the significant GO terms enriched in the high-risk group. (**b**) GSEA of the significant KEGG pathways enriched in the high-risk group.

To explore the differences in biological functions between the high-risk and low-risk groups based on risk score, we used GSEA software to analyze GO terms and KEGG pathways throughout the complete dataset. In the high-risk group, the significantly enriched GO terms were associated with cellular response to osmotic stress, endosomal transport, regulation of RAS protein signal transduction, transmembrane receptor protein kinase activity, *et al.* In the high-risk group, significant enrichment pathways included ECM receptor interaction, focal adhesion, MAPK signaling pathway, *et al.* The details of the GO terms and KEGG pathway are provided in Table **S3** (Fig. **7a-b**).

### 3.6 Expressions of 14 NRlncRNAs in OC Cell Lines

**Fig. (8):**
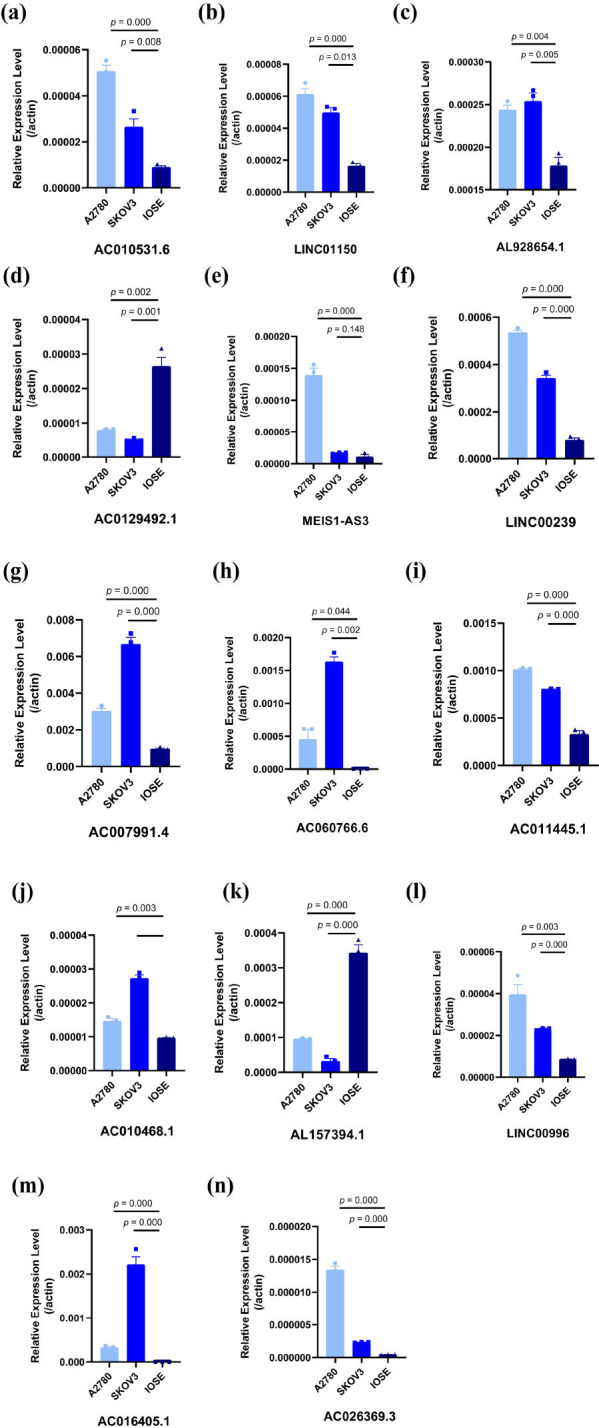
The qRT-PCR results of 14 NRlncRNAs in two OC cell lines and normal ovary cell lines. (**a**) *AC010531.6* (**b**) *LINC01150* (**c**) *AL928654.1* (**d**) *AC129492.1* (**e**) *MEIS1-AS3* (**f**) *LINC00239* (**g**) *AC007991.4* (**h**) *AC060766.6* (**i**) *AC011445.1* (**j**) *AC010468.1* (**k**) *AL157394.1* (**l**) *LINC00996* (**m**) *AC016405.1* (**n**) *AC026369.3.*

To validate the expression pattern of the 14 identified NRlncRNAs, qRT-PCR was conducted on two OC cell lines (A2780 and SKOV3) and a normal ovarian cell line (IOSE). The results confirmed that the expressions of *AC010531.6*, *LINC01150*, *AL928654.1*, *MEIS1-AS3*, *LINC00239*, *AC007991.4*, *AC060766.6*, *AC011445.1*, *AC010468.1*, *LINC00996*, *AC016405.1*, and *AC026369.3* were upregulated in OC cell lines and that *AC129492.1* and *AL157394.1* were downregulated, consistent with the results from the TCGA and GTEx (Fig. **8a-n**). However, the expression of *MEIS1-AS3* was not higher in SKOV3 than in IOSE cells, which may be attributable to the different cancer cell lines.

## 4 DISCUSSION

Necroptosis differed from cell apoptosis, which was considered to restrain tumor development. However, necroptosis was increasingly recognized to contribute to the regulation of the cancer biology process, including tumor formation, tumor metastasis, and immunity [38]. Boris *et al.* reported that necroptosis played an essential role in triggering inflammatory responses and promoting cancer metastasis and immunosuppression [39]. VDX-111, a novel small molecule, induced necroptosis to inhibit ovarian cancer progression [40]. Ilana *et al.* found that a pan-ALDH1A inhibitor-induced necroptosis in OC stem-like cells [41]. Zhang *et al.* revealed that ceramide nanoliposomes can serve as an MLKL-dependent, necroptosis-inducing, chemotherapeutic reagent in OC [42]. Zheng *et al.* demonstrated that RIP1 promoted proliferation through G2/M checkpoint progression and mediated cisplatin-induced apoptosis and necroptosis in human OC cells [43]. Wu *et al.* showed progesterone prevented OC by inducing necroptosis of p53-defective fallopian tube epithelial cells [44]. Chen *et al.* uncovered CuS-MnS2 nano-flowers for magnetic resonance imaging-guided photothermal/photodynamic therapy of OC through necroptosis [45]. Hernandez *et al.* determined a dual role for Caspase8 and NF- κ B interactions in regulating apoptosis and necroptosis of OC [46]. Thibault *et al.* confirmed that DEBIO 1143 was an IAP inhibitor that reversed carboplatin resistance in OC cells and triggered apoptotic or necroptotic cell death [47]. Several lncRNAs have been reported to regulate necroptosis through function as competitive RNAs. A deeper understanding of the NRlncRNAs was needed to improve the unsolvable difficulties in OC therapy. This research effort led to the development of a new NRlncRNAs model to predict clinical outcomes and immunotherapy responses based on bioinformatics analysis in OC.

Firstly, we collected the NRGs profiles from the TCGA and GTEx and obtained 30 NRlncRNAs through co-expression, different expression, and univariate Cox. Then, the LASSO and multivariate Cox methods were conducted to establish a 14 NRlncRNAs model containing *AC010531.6*, *LINC01150*, *AL928654.1*, *AC129492.1*, *MEIS1-AS3*, *LINC00239*, *AC007991.4*, *AC060766.6*, *AC011445.1*, *AC010468.1*, *AL157394.1*, *LINC00996*, *AC016405.1* and *AC026369.3* for OC. According to RNAct database (https://rnact.crg.eu/) [48], we can classify our selected lncRNAs according to biotype, *AC010531.6*, *AC129492.1*, *MEIS1-AS3*, *AC007991.4*, *AC026369.3* were antisense RNA; *LINC01150*, *LINC00239*, *AC060766.6*, *LINC00996* were lincRNA; *AL928654.1*, *AC010468.1* were processed pseudogene; *AC016405.1* was sense intronic; *AC011445.1*, *AL157394.1* were sense overlapping. Additionally, we validated the model on the train, test, and entire datasets. The risk score has the potential to be an independent prognostic factor in OC. In addition, the significant implication of the NRlncRNAs model in OC was the contribution to the prediction of immunotherapy response. Of all the treatment strategies, the most promising treatment was the immune-related treatment of cancer. In the last few years, advances in immunotherapy have made it possible for patients to live longer than in the past in several types of cancers. However, there was no denying the fact that how to determine the eligibility of patients was an undefined issue. The low-risk group of patients showed elevated scores in most immune cells and pathways and a reduced TIDE score, indicating a reduced likelihood of immune escape. Consequently, we hypothesized that low-risk OC patients could be better candidates for immunotherapy.

Additionally, clinical experiments have demonstrated that TMB levels were highly consistent with the activity of immune checkpoint blockers [49]. The accumulating evidence revealed that a higher TMB level was a good guide to a better response to immunotherapy treatment [50]. The results of this study demonstrated that the low-risk group had elevated TMB and neoantigen numbers compared to the high-risk group, implying that the low-risk subtype could be more responsive to immune checkpoint therapy due to the higher TMB levels. Notably, another significant implication of the NRlncRNAs model in OC was the prediction for target drugs PARP inhibitors. As we know, OC patients with HRD displayed distinct clinical characteristics and responded more favorably to platinum-based chemotherapy and PARP inhibitors. Our analysis revealed significant differences in HRD status in the high-risk and low-risk groups. The low-risk patients processed a significantly higher level of HRD score, which implied a better response for PARP inhibitors. More importantly, by comparing with dozens of previously published prognostic risk models, our model has superior AUC at the 1-year, 3-year, and 5-year intervals, including the model of necroptosis-related lncRNAs previously published in OC. Above all, the risk model may aid us in conducting individualized treatment plans involving the compatibility of immunotherapy and targeted therapy.

Some lncRNAs included in the model have previously been shown to play crucial roles in various cancers. *AC026369.3* was shown to be involved in autophagy, N6-Methyladenosine, and TME, as well as patients’ clinical outcomes in several carcinomas [51-54]. *AC010531.6* was reported as a prognostic ferroptosis-related lncRNA in gastric cancer [55], and it was also shown to be linked with immune in epithelial OC [56]. Zhang and colleagues [57] found *AC007991.4* combined with another two lncRNAs served as an independent predictor for gastric cancer prognosis. *AC011445.1* was presented to be pivotal in the process of ferroptosis and immune-associated ovarian carcinoma [58]. *MEIS1-AS3* was a component of the autophagy-related lncRNA signature in OC patients [59]. *AL157394.1*, an m6A-related lncRNA, has demonstrated prognostic importance in kidney renal clear cell carcinoma cases [60]. As mentioned above, these lncRNAs have garnered increasing attention due to their essential roles in numerous biological processes, such as cancer progression, immune response, and tumor formation.

## 5 STUDY LIMITATIONS

However, this study had several limitations. Even though we examined the expressions of the 14 NRlncRNAs in OC cell lines and normal ovary cell lines, more experiments were still required to investigate the regulatory roles of these 14 lncRNAs on necroptosis in OC. Furthermore, future studies should include an expanded cohort of OC samples to validate the reliability and effectiveness of the 14 NRlncRNAs model.

## CONCLUSION

Overall, this study provided a comprehensive knowledge base of new NRlncRNAs in OC, contributing to a deeper understanding of lncRNA roles in regulating necroptosis in OC. We established a novel NRlncRNAs model as a prognostic indicator for predicting survival outcomes, immunotherapy, and PARP inhibitors response in OC. The performance of our model surpassed many models previously reported in the literature. Our findings may provide convincing evidence to guide personalized treatment plans for OC patients in clinical settings.

## Figures and Tables

**Fig. (1):**
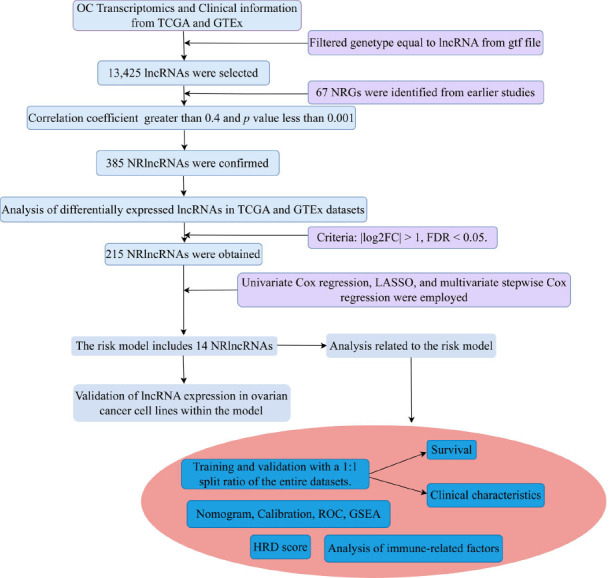
The study’s flowchart.

## Data Availability

The data sets generated and/or analyzed during the current study were not publicly available but were available from the corresponding author on reasonable request.

## LIST OF ABBREVIATIONS

GSEA: Gene Set Enrichment Analyses
NRGs: Necroptosis-Related Genes
OC: Ovarian Cancer
RIPK3: Receptor-Interacting Protein Kinase 3
