# Supplementary material for: Establishment of a Prognostic Necroptosis-Related lncRNA Signature in Ovarian Cancer
Source: Comb Chem High Throughput Screen. 2025 Jan 7;29(4):577–94. doi: 10.2174/0113862073339602241028095015 (PMC13312397; doi:10.2174/0113862073339602241028095015)
Supplement: Supplementary file 1 [file CCHTS-29-4-577_SD1.pdf]

Supplementary Material

Establishment of a Prognostic Necroptosis-Related lncRNA Signature in Ovarian Cancer

Hui Xu[1], Meng Li[1], Wen-lan Qiao[1] and Tian Hua[1,*]

[1]*Department of Gynecology, Affiliated Xingtai People Hospital of Hebei Medical University, China*

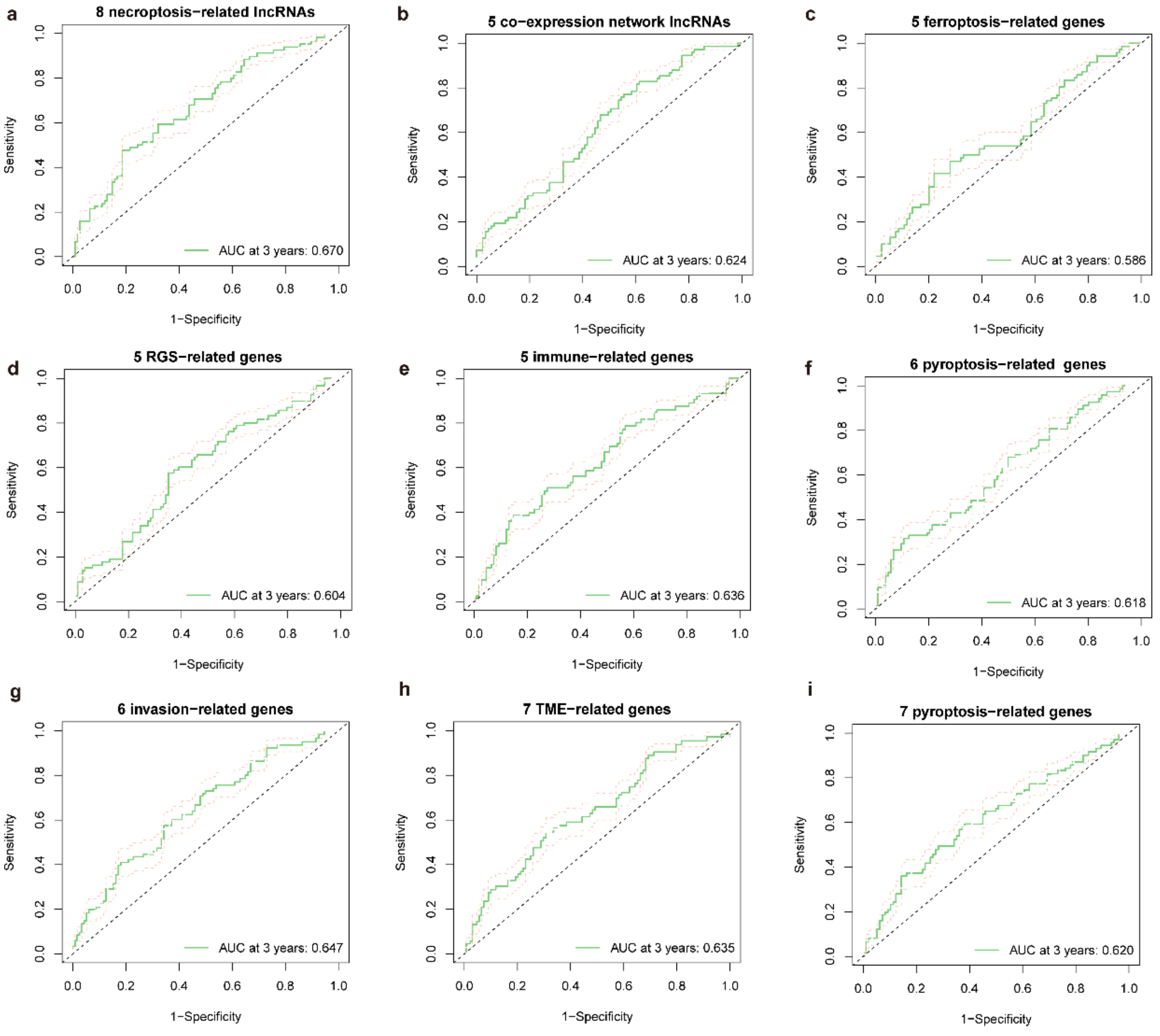

Fig. (S1). The ROC plots of compared prognosis models.

Table 1 The primer sequences of necroptosis-related lncRNAs (NRlncRNAs).

LncRNA	The Primer Sequences
AC010531.6	AGGGTCTCGGTAGCCTGCAA
	AGAGCTTCGGTGCTTGCACG
LINC01150	AGTCGCTCTGGTTCACACGC
	GTCTGGCGGGGATATTTCAT
AL928654.1	CCACCTGTCAGGATGCCTTC
	TGATAGCCCTGAACGCTCCG
AC129492.1	GTGGGGATGCCCTCCATGAT
	TACCTCAACGGCGTCAACCC
MEIS1-AS3	AGCTGCGGACTGAACCCTCA
	CTCGCATAAATCGCCTGAAC
LINC00239	GAACGGAGGCGTTTCAGGCA
	TCCAATGAGGCAGGTGTGCG
AC007991.4	TGTATTTTTAGTAGAGACGAGG
	ATAAAGAAAATATGCCCTGG
AC060766.6	ATATCCAGGGGCTAATATAA
	TTTTTAACTGCACCTGTTTG
AC011445.1	GGCTTTCTCATCGCGCTGTT
	CCCGTTTATTGACAGCCTCC
AC010468.1	AGGCAAGATCAAAAGGCTGT
	TTTTACTGCGACCAACATTA
AL157394.1	ATAACATTTTTTGGGGTGGGTG
	TTAACCTGCTATCCACTGGC
LINC00996	TTTCAGTGGCCCTCGTGGAA
	CGCTGCCAACTGCTAAGATG
AC016405.1	AGGAGAGTGACCTGTCAGAT
	TCCCATCTCTCTGTTATGAT
AC026369.3	CTCACGCATGAGCCAAGATA
	TTTTCTCACTTCCAGGAAGA

Table S2. The profile of significantly different immune cell infiltration between risk groups across various platform.

Immune_Cell	Cor	*p*_value
B cell_TIMER	-0.204	0.001
Macrophage_TIMER	0.183	0.003
B cell plasma_CIBERSORT	-0.219	0
T cell CD4+ memory activated_CIBERSORT	-0.139	0.024
T cell follicular helper_CIBERSORT	-0.287	0
Macrophage M1_CIBERSORT	-0.206	0.001

Macrophage M2_CIBERSORT	0.17	0.006
Myeloid dendritic cell activated_CIBERSORT	-0.125	0.043
Mast cell resting_CIBERSORT	0.162	0.008
B cell plasma_CIBERSORT-ABS	-0.246	0
T cell CD4+ memory activated_CIBERSORT-ABS	-0.139	0.024
T cell follicular helper_CIBERSORT-ABS	-0.295	0
NK cell activated_CIBERSORT-ABS	-0.123	0.045
Macrophage M1_CIBERSORT-ABS	-0.171	0.005
Mast cell resting_CIBERSORT-ABS	0.145	0.018
Macrophage M1_QUANTISEQ	0.126	0.041
T cell regulatory (Tregs)_QUANTISEQ	-0.13	0.035
NK cell_MCPCOUNTER	-0.129	0.036
Neutrophil_MCPCOUNTER	0.146	0.017
Endothelial cell_MCPCOUNTER	0.131	0.034
Cancer associated fibroblast_MCPCOUNTER	0.225	0
T cell CD8+_XCELL	-0.136	0.027
T cell CD8+ central memory_XCELL	-0.227	0
Class-switched memory B cell_XCELL	-0.161	0.009
Cancer associated fibroblast_XCELL	0.214	0
Hematopoietic stem cell_XCELL	0.161	0.009
Plasmacytoid dendritic cell_XCELL	-0.24	0
B cell plasma_XCELL	-0.138	0.025
T cell gamma delta_XCELL	-0.149	0.015
T cell CD4+ Th1_XCELL	-0.187	0.002
T cell CD4+ Th2_XCELL	-0.174	0.005
stroma score_XCELL	0.212	0.001
B cell_EPIC	-0.148	0.016
Cancer associated fibroblast_EPIC	0.213	0
T cell CD8+_EPIC	-0.125	0.042
uncharacterized cell_EPIC	-0.201	0.001

Table S3. The details of GSEA analysis.

Term	*p* value	FDR	NES
KEGG_ECM_RECEPTOR_INTERACTION	0	0.009	2.13
KEGG_FOCAL_ADHESION	0	0.013	2.18
KEGG_MAPK_SIGNALING_PATHWAY	0	0.012	2.06
KEGG_MELANOMA	0	0.019	1.98
KEGG_MTOR_SIGNALING_PATHWAY	0.001	0.01	2.09
KEGG_SMALL_CELL_LUNG_CANCER	0	0.019	1.99
